# Review of Stereo Matching Algorithms Based on Deep Learning

**DOI:** 10.1155/2020/8562323

**Published:** 2020-03-23

**Authors:** Kun Zhou, Xiangxi Meng, Bo Cheng

**Affiliations:** ^1^School of Mathematics Science, Peking University, Beijing, China; ^2^Suzhou Automotive Research Institute, Tsinghua University, Beijing, China; ^3^Key Laboratory of Carcinogenesis and Translational Research (Ministry of Education/Beijing), Department of Nuclear Medicine, Peking University Cancer Hospital & Institute, Beijing, China; ^4^Su Zhou Automobile Research Institute, Suzhou, Jiangsu, China

## Abstract

Stereo vision is a flourishing field, attracting the attention of many researchers. Recently, leveraging on the development of deep learning, stereo matching algorithms have achieved remarkable performance far exceeding traditional approaches. This review presents an overview of different stereo matching algorithms based on deep learning. For convenience, we classified the algorithms into three categories: (1) non-end-to-end learning algorithms, (2) end-to-end learning algorithms, and (3) unsupervised learning algorithms. We have provided a comprehensive coverage of the remarkable approaches in each category and summarized the strengths, weaknesses, and major challenges, respectively. The speed, accuracy, and time consumption were adopted to compare the different algorithms.

## 1. Introduction

**Figure 1:**
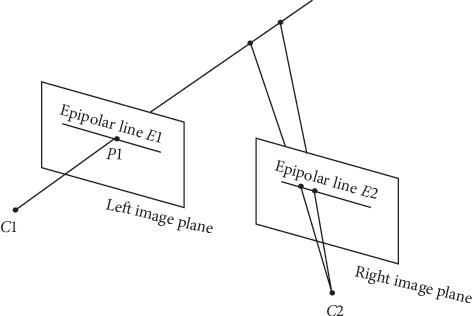
Geometry of epipolar lines, where *C*1 and *C*2 are the left and right camera lens centers, respectively. Point *P*1 in one image plane may have arisen from any of the points in the line *C*1*P*1 and may appear in the alternate image plane at any point on the epipolar line *E*2.

Stereo disparity estimation is one of the most important problems in computer vision. The disparity map has a wide range of applications, including robotics [1], object detection [2], remote sensing [3], and autonomous driving [4]. Finding corresponding pixels from two viewpoints is the key point of stereo matching, which is similar to optical flow estimation. Owing to the epipolar constraint based image rectification, the search space for the matching can be limited to a 1D horizontal line, as compared to a 2D plane in optical flow [5]. Namely, depth can be estimated by matching corresponding pixels on the two rectified images along the same scan line. As shown in Figure 1, a point *P*1 in one image plane may have arisen from any of the points in the line *C*1 *P*1 and may appear in the alternate image plane at any point on the so-called epipolar line *E*2. Thus, the search is theoretically reduced within a scan line, since corresponding pair points reside on the same epipolar line. The difference on the horizontal coordinates of these points is the disparity. Then the depth of this pixel is calculated by *fB*/*d*, where *f* is the camera's focal length and *B* is the distance between two camera centers.

**Figure 2:**
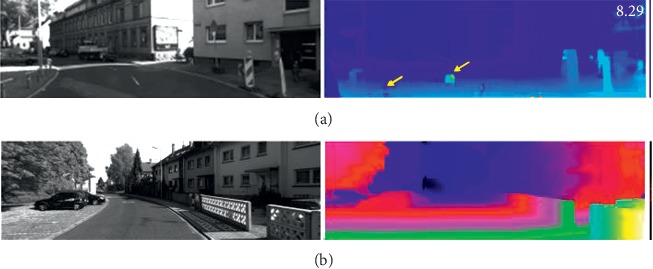
Depth map comparison between SGM (a) and GC-Net (b).

As a classical research topic for decades, stereo matching was traditionally formulated as a multistage optimization problem [6, 7], including matching cost computation, cost aggregation, disparity optimization, and postprocessing [8]. Matching cost computation is the first step of stereo matching, which provides initial similarity measurements for left image patches and possible corresponding right image patches. Traditional stereo matching methods usually utilize the low-level features of image patches around the pixel to measure the dissimilarity. Some common local descriptors, such as absolute difference (AD), CENSUS [9], BRIEF [10], normalized cross-correlation (NCC) [11], or their combinations (e.g., AD-CENSUS), are often employed. The cost aggregation and optimization steps incorporate contextual matching costs and regularization terms to obtain more robust disparity predictions. Traditional stereo matching algorithms can be grouped into three categories: (1) local methods, (2) global methods, and (3) semiglobal methods. Local methods are done by selecting the disparity with the lowest matching cost, that is, the “winner takes all” strategy. It runs very fast but suffers from low quality. Some global methods, such as graph cut [12] or belief propagation [13], skip the cost aggregation step and define a global energy function. The disparity is obtained with a high quality by minimizing the energy function step by step; however, the method is time consuming. Semiglobal methods [6] approximately solve the NP-hard 2D graph partitioning by optimizing a pathwise form of the energy function in many directions. This method achieves a fair trade-off between the complexity of the computations needed and the quality of the results obtained. However, performance of the traditional stereo matching methods is severely limited by the handcrafted features adopted by cost functions. As shown in Figure 2, it is obvious that the traditional SGM methods suffer from poor depth map quality compared to the GC-Net, which is an end-to-end deep learning method. The SGM-method suffers from obvious bad pixels while the GC-Net provides a much smoother and more consistent depth map. A detailed review of the traditional stereo matching algorithms could be found in [14, 15]. In this article, we will focus on the algorithms based on deep learning.

Recently, stereo matching algorithms have become a deep learning task resorting to the development of convolutional neural networks (CNN). For convenience, we classify the algorithms into three categories: (1) non-end-to-end learning algorithms, (2) end-to-end learning algorithms, and (3) unsupervised learning algorithms. For non-end-to-end stereo methods, CNN is introduced to substitute one or more components in the legacy stereo pipeline. Zbontar and LeCun [16] first successfully substituted handcrafted matching cost metrics with deep metrics and achieved considerable gain compared to traditional approaches in terms of both accuracy and speed. They introduced a deep Siamese network to measure the similarity between two 9-by-9 image patches. Later, Luo et al. [17] accelerated matching cost calculation by introducing an inner-product layer and treated the patch matching as multilabel classification problem. Shaked et al. [18] designed a new highway network architecture for computing the matching cost at each possible disparity. Chen et al. [19] employed multiscale features for matching cost calculation. Some works focus on the cost aggregation and postprocessing unit. Seki and Pollefeys [20] proposed SGM-Nets to provide learned penalties for SGM. Knobelreiter et al. [21] learned smoothness penalties through a CRF and combined it with a CNN-predicted correlation matching costs to integrate long-range interactions. Gidaris and Komodakis [22] substituted handcrafted disparity refinement functions with a three-stage network that detects, replaces, and refines erroneous predictions.

All these methods have achieved great gains, compared with the traditional ones. However, limitations of these stereo networks are obvious [23]: (1) high computational burden from multiple forward passes for all potential disparities; (2) limited receptive field and the lack of context information to infer reliable correspondences in ill-posed; (3) still using postprocessing functions which are hand-engineered with a number of empirically set parameters.

By carefully designing and supervising the network, a fine disparity could also be obtained by end-to-end deep learning methods without postprocessing. With the success of Mayer et al. [24], end-to-end stereo matching networks become more and more popular in stereo matching algorithms. Tons of algorithms based on this have been proposed. These methods could roughly be categorized into two groups: 2D encode-decoder structures [23–27] and regularization modules composed of 3D convolutions [28–31]. DispNetC [24] computes a correlation volume from the left and right image features (encoding) and utilizes a CNN to directly regress (decoding) disparity maps. Pang et al. [26] proposed a two-stage architecture called cascade residual learning (CRL), and each of the stages adopts the DispNet structure. The first stage gives initial predictions, and the second stage learns the residuals. Liang et al. [23] extended DispNet and designed a different disparity refinement subnetwork, in which two stages are combined for joint learning based on the feature constancy. Xiao et al. [32] proposed a network composed of a backbone disparity network and an edge subnetwork to integrate additional information. GC-NET [29] first employed 3D convolution module to regularize the cost volume and incorporate more context from the disparity dimension. Inspired by GC-Net, Chang and Chen [28] employed a spatial pyramid pooling module to extract multiscale representations and incorporate a stacked 3D CNN to aggregate contextual features. Lu et al. [5] proposed the sparse cost volume net (SCV-Net) based on GC-Net to achieve speed acceleration. Some works focus on designing specific functional modules. Lidong et al. [30] proposed a learning-based cost aggregation method for the better generation and selection of cost aggregation. Jie et al. [33] proposed a novel left-right comparative recurrent (LRCR) model to perform left-right consistency checking jointly with and end-to-end disparity estimation.

Modern deep learning-based algorithms are able to generate highly accurate depth estimates from stereo image pairs. However, state-of-the-art stereo methods still have difficulties finding correct correspondences in textureless regions, detailed structures, small objects, and near boundaries. Moreover, end-to-end stereo matching networks-based approaches basically require huge memory and are relatively time consuming. And of course, this kind of end-to-end stereo matching network needs corresponding ground truth depth data for training, which means a huge amount of work of data labeling.

Over the past few years, based on spatial transformation and view synthesis, several unsupervised learning methods have been proposed for stereo matching [34–38]. The Deep3D network [37] involves an unsupervised framework to address the problem of novel view synthesis. It can generate the corresponding right view from an input left image, i.e., the reference image. Garg et al. [35], like in Deep3D, trained a network for depth estimation using a not fully differentiable image reconstruction loss derived from Taylor expansion. Godard et al. [36] extended the image reconstruction loss by using bilinear sampling to generate images, resulting in a fully differentiable training loss. The loss also incorporated consistency between the disparities produced relative to both the left and the right images, leading to improved performance and robustness, making it a popular end-to-end unsupervised structure. Based on this architecture, Zhong et al. [39] proposed an unsupervised self-adaptive stereo matching network combining two GC-Nets together. Smolyanskiy et al. [40] slightly changed the architecture and proposed a semisupervised approach. Other methods focusing on the optical flow estimation by incorporating the pose information [38, 41] have also been studied. However, extending these monocular methods to stereo matching is nontrivial. To date, unsupervised depth solutions, while yielding encouraging preliminary results, are still not at the point where reliable information can be expected from.

To assist future researchers in developing their own stereo matching algorithms, we herein provide a comprehensive coverage of the top approaches belonging to these three kinds of algorithms. The performance of these algorithms such as speed, accuracy, time consumption, was analyzed and compared with each other. The whole comparison is conducted based on the KITTI datasets including KITTI 2012 and KITTI 2015. The performance comparison of stereo matching framework is listed in Table 1, including the advantage and disadvantage of each framework.

The KITTI stereo dataset is a collection of grayscale image pairs taken from two video cameras mounted on the roof of a car, roughly 54 centimeters apart. The images are recorded while driving in and around the city of Karlsruhe, in sunny and cloudy weather, at daytime. It consists of KITTI2012, which contains 194 stereo pairs at the resolution of 1240 × 376 for training with sparse ground truth disparities and 195 testing pairs without ground truth, and KITTI2015, which contains 200 training pairs and 200 testing pairs. Each image pair is rectified, i.e., transformed in such a way that an object appears on the same vertical position in both images. A rotating laser scanner, mounted behind the left camera, provides ground truth depth. The true disparities for the test set are withheld, and an online leaderboard is provided where researchers can evaluate their method on the test set.

All the performance data of these methods is listed in several tables to provide a comprehensive comparison. The evaluation metric is usually the end-point error (EPE), which is the mean average disparity error in pixels. For KITTI 2012, percentages of erroneous pixels and average end-point errors for both non-occluded (Non-occ) and all (All) pixels are reported. For KITTI2015, the percentage of disparity outliers D1 is evaluated for background, foreground, and all pixels. The outliers are defined as the pixels whose disparity errors are larger than max (3px, 0.05d∗), where d∗ denotes the ground truth disparity. The performance of the unsupervised methods is listed in Table 2,where the absolute relative error (Abs Rel), square relative error (Sq Rel), root mean square error (RMSE), and the *δ* < 1.25 error provide a comprehensive comparison among the methods. The *δ* < 1.25 error means the percent of pixels that satisfy *δ* < 1.25, where *δ* is calculated by the following equation:

(1) $$\begin{array}{c} \delta =\max\left(\frac{{\text{Disp}}_{\text{pred}}}{{\text{Disp}}_{\text{gt}}},\frac{{\text{Disp}}_{\text{gt}}}{{\text{Disp}}_{\text{pred}}}\right), \end{array}$$

where the Disp_gt_ means the ground truth disparity and Disp_pred_ means the predicted disparity.

## 2. Non-End-To-End Stereo Matching

**Figure 3:**
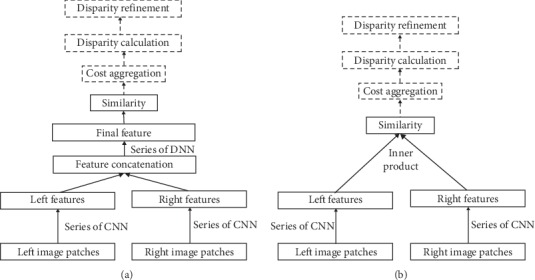
Two Siamese network structures: (a) the basic Siamese network structure to estimate the similarity between two image patches; (b) the accelerated Siamese network by employing a dot layer.

For non-end-to-end stereo methods, CNN has been introduced to replace one or more components in the legacy stereo pipeline. Zbontar and LeCun [16] first proposed to compute matching costs using neural networks and named it MC-CNN. A deep Siamese network composed by several CNN and DNN layers was adopted to measure the similarity between two 9-by-9 image patches, as shown in Figure 3(a). Then, the similarity, i.e., the cost, was refined by cross-based cost aggregation and semiglobal matching, followed by a left-right consistency check to eliminate errors in the occluded regions. This method achieved the state-of-the-art results on the KITTI stereo dataset. The success of this method firmly demonstrated that the image features extracted by CNN are much more precise compared to the handcrafted ones. As a consequence, many of the other top ranked methods either are inspired by MC-CNN or directly use it to compute the matching cost [22, 43–48]. Zagoruyko and Komodakis [49] explored and proposed a variety of different neural network models (Siamese, pseudo-Siamese, 2-channel) to represent the similarity function that accounts for a broader set of appearance changes and can be used in a much wider and more challenging set of applications. The conclusions are obvious and intuitive: (1) the more complex the network, the better the performance; (2), the larger the training dataset, the better the performance.

While these methods [16, 49] achieved great gains in challenging benchmarks such as KITTI [50], they suffer from high time consumption due to the fact that they exploit a Siamese architecture followed by concatenation and further processing via a few more fully connected layers (DNN) to compute the final score [17], as shown in Figure 3(a). For instance, suppose the image size is M-by-N, the max disparity is *D*, and the inference time of the Siamese network is *T*; it will take *M* ∗ *N* ∗ (*D* + 1) ∗ *T* to conduct the cost computation step. Therefore, if *T* is very large, the time consumption will become unbearable. It took 67s for the MC-CNN [16] method to predict a single image pair (KITTI data, 1226 *∗* 370), which is far beyond a practical expectation. To address this problem, Chen et al. [19] proposed an embedding model fusing multiscale features in matching cost calculations. Given the feature vectors (corresponding to the left-right patches in a stereo pair) output by the CNN, the similarity was directly computed in the Euclidean space by a dot product, as shown in Figure 3(b). In these methods [16, 49], feature vectors require further fully connected DNN to obtain the final similarity. This change achieves 100x speed-up compared to MC-CNN [16]. Luo et al. [17] also employed an inner-product layer and proposed to learn a multilabel classification model over all possible disparities. The inner-product layer greatly decreases the computation burden while the multilabel classification thought enhances the matching performance. This is because the multilabel model is able to capture correlations between the different disparities implicitly by learning a probability distribution over all disparity values using a smooth target distribution.

In these approaches, several unlearned postprocessing functions are followed after obtaining the cost volume through CNN, including cross-based cost aggregation, semiglobal matching, left-right consistency check, subpixel enhancement, and filtering. The performance of the approaches [16, 17, 19] is listed in Table 3 to give a comprehensive exploration of the methods employing CNN to replace handcrafted features. The OCV-SGBM provided by the OpenCV community was introduced as a standard, because all the other methods share the same postprocessing functions except for the cost calculation step. The OCV-SGBM adopts the handcrafted features while the other methods exploit the CNN-based features. From Table 3, we can find that the CNN-based features greatly improved the accuracy; however, they also greatly increased the time consumption. The Siamese network could greatly improve the performance of the cost computation step, making it more precise; however, it needs much more computation resources compared to the handcrafted feature SAD, which is adopted by OCV-SGBM. It is noted that the other methods are conducted on the Nvidia Titan X while the OCV-SGBM is conducted only on CPU at 2.5 GHz, which means that the standard OCV-SGBM is approximately 100x faster than the other methods listed in Table 3.

Of course, there are some other researchers focusing on designing a more complex network to solve the patch matching problem, because the original simple convolutional layers are limited to generate rich semantic representations. As Zagoruyko and Komodakis [49] has already proved that the more complex networks could enhance the model performance, these kinds of work could present us some new designs of the network. Park and Lee [51] proposed a per-pixel pyramid pooling layer which can cover a large area without losing resolution or details to enlarge the perception window size. Shaked et al. [18] designed a new highway network architecture for computing the matching cost at each possible disparity based on multilevel weighted residual shortcuts. All these methods focus on the calculation of the cost and achieve great gain in performance compared to traditional algorithms.

Deep neural networks could also be employed to substitute other components in the legacy stereo pipeline. Based on the observation that disparity images are generally piecewise smooth, some existing works impose smoothness constraints in the learning process. Seki and Pollefeys [20] raised the SGM-Net framework that predicts SGM penalties for regularization. It takes a gray scale image patch of 5 × 5 pixels and its normalized position as input and then gives the prediction of SGM penalties. A novel loss consisting of path cost and neighbor cost was introduced in this network to enable the usage of sparsely annotated disparity maps such as the ones captured by a LiDAR sensor in real environments. The SGM-NET achieves the state-of-the-art accuracy on KITTI benchmark datasets. However, due to the fact that SGM penalties could not be labeled explicitly, the network has to employ a three-step procedure to generate weak labels of the SGM penalties for training, making the whole process complicated and time consuming.

Knobelreiter et al. [21] learned smoothness penalties through a hybrid CNN + CRF model for energy function optimization. Unary-CNN and pairwise-CNN were used to extract expressive features, based on which unary cost and binary cost of CRF were calculated. A theoretically sound method based on the structured output support vector machine (SSVM) was proposed to train the hybrid CNN + CRF model on large-scale data end-to-end. This method achieved comparable results compared to the state-of-the-art methods. This method is similar to the traditional global methods such as GC (graph cut) and belief propagation. However, in traditional global methods, the features are known and the disparity is calculated by iteratively minimizing the energy function composed of features and disparity, while in this method, the feature is unknown and the disparity is known, so the feature could be calculated by the SSVM and then work as the label to train the CNN + CRF network.

Some studies focus on the postprocessing of the disparity map. Gidaris and Komodakis [22] substituted handcrafted disparity refinement functions with a three-stage network that detects, replaces, and refines erroneous predictions. The network architecture improves the labels by detecting incorrect labels, replacing them with new ones, and refining the renewed labels (DRR). Based on this three-stage structure, they achieved state-of-the-art results in the KITTI 2015 test. However, discarding unreliable disparities with new ones resulted in a wasted computation resource. The Displets [52] aims to address the problem that reflective and textureless surfaces cannot be recovered easily using traditional local regularizers. The method was proposed based on the fact that objects generally exhibit regular structures and are not arbitrarily shaped. In the Displets method, regularization over larger distances using object-category specific disparity proposals, i.e., Displets, is used to resolve matching ambiguities in reflective and textureless regions. This approach embeds a 3D model of vehicles and ranks first across all KITTI stereo leaderboards. However, the introduction of the models greatly improves the computation burden, as shown in Tables 4 and 5.

These non-end-to-end methods, a number of handcrafted regularization functions or postprocessing stages, are still necessary to achieve comparable results. And they may suffer from high computational burden, limited receptive field, and lack of context information and still use postprocessing functions more or less. As is explicitly demonstrated in Tables 4 and 5, all the methods achieved great performance while suffering from the time consumption. DDR [22] achieved the best time performance due to the fact that the network was designed for the whole image while the networks in other methods were designed for image patch. As a result, the DDR method only needs one time calculation of the network while other methods need *M* ∗ *N* (*M* is the number of rows of the image, *N* is the number of columns of the image) times of calculations of the network.

## 3. End-To-End Stereo Matching

**Figure 4:**
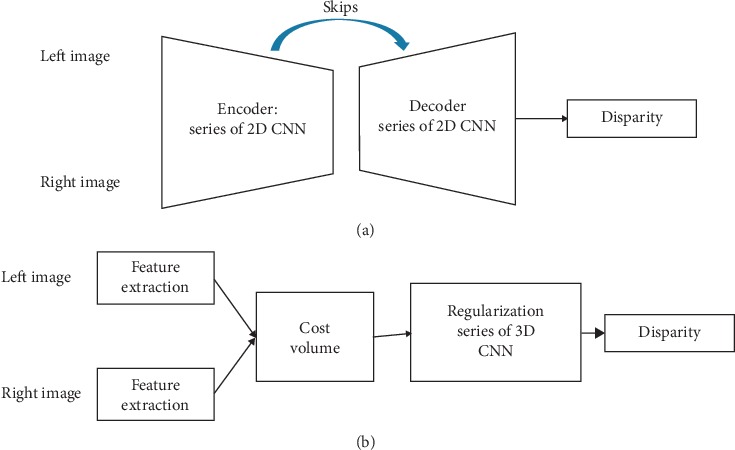
The two popular basic architectures for end-to-end disparity estimations: (a) 2D encoder-decoder structure; (b) 3D regularization structure.

The end-to-end disparity estimation networks seamlessly integrate all steps in the stereo matching pipeline for joint optimization [24], producing dense disparity maps from stereo images directly. Since the first success of Mayer [24], end-to-end stereo matching networks have become more and more popular in stereo matching algorithms. A lot of algorithms based on this have been proposed. 2D encoder-decoder structures with cascaded refinement and regularization modules composed of 3D convolutions are two most popular structures among current end-to-end stereo matching networks. As shown in Figure 4(a), 2D encoder-decoder structure is composed of a series of stacked 2D CNN with some skips to bring detailed or, in other words, residual information for the final prediction, thus improving the performance. The key point of the 3D structure is the exploit of the disparity dimension by using 3D CNN.

Critically speaking, Dosovitskiy et al. [53] are the first to employ an end-to-end network to solve the stereo matching problem. Appropriate end-to-end CNN including FlowNet and FlowNetC have been proposed to solve the optical flow estimation problem. The FlowNet provides the basic 2D encoder-decoder structure. Later, a lot of networks [23, 24, 26, 27, 32] have been proposed based on this. Optical flow estimation requires precise per-pixel localization, and it also depends on finding correspondences between two input images. The critical difference between optical flow estimation and stereo matching is the search space. Owing to the epipolar constraint, the search space for the matching can be limited to the 1D horizontal line, as compared to a 2D search in optical flow. Thus, technically speaking, the solution to the optical flow [53–59] problem could be easily applied to the stereo matching problem with a bit change.

Inspired by FlowNet [53], Mayer et al. [24] proposed DispNet, which combines a flow and a disparity estimation network together. A 1D correlation layer along the disparity line was proposed for the cost calculation, and an encoder-decoder structure with shortcut connections was designed for disparity regression. This method became the first end-to-end network for the disparity estimation and reaches the state-of-the-art results in disparity estimation. The end-to-end structure makes the disparity estimation problem much easier. All you need to do is designing a network which takes the image pair as input and predicts the disparity directly. And as the network takes the entire image as input, it is much more efficient compared to Siamese network adopted by the non-end-to-end methods. As shown in Table 6, the speed of DispNet is much faster. Despite this, it is still difficult to find the correct correspondence at inherently ill-posed regions, such as object occlusions, repeated patterns, or textureless regions. Therefore, a lot of works have been focused on addressing this problem by verifying DispNet.

Inspired by DispNet, Pang et al. [26] proposed a two-stage architecture called cascade residual learning (CRL) where the first stage gives initial predictions, and the second stage performs further refinement/rectification by producing residual signals across multiple scales. The main structures of both stages share similar spirits with DispNetC [24] and the summation of the outputs from the two stages gives the final disparity. The more complex structure results in a more powerful representation capability, and the two-stage architecture is beneficial to capturing the refinement information. As a result, this method achieved a great performance improvement and reached state-of-the-art performance for matching stereo correspondence. However, the more complex structure means a higher calculation burden, and as a consequence, this method is 8x slower than DispNet, as shown in Table 7.

Liang et al. [23] extended DispNet and designed a different disparity refinement subnetwork, in which two stages are combined for joint learning based on the feature constancy. This method incorporates all the four steps of stereo matching together. The adoption of feature correlation and reconstruction error makes the network easy for optimization. This architecture achieved a great performance gain with only a little sacrifice of the speed compared to DispNet. The CRL [26] method and iResNet [23] share similar thoughts. One network predicts the initial disparity, while the other predicts the residual. However, the CRL method does not share sufficient information between the two subnetworks. Only the disparity information predicted by the first stage subnetwork was passed to the second stage subnetwork, while the iResNet [23] shares much more information between the two subnetworks. This is the main reason that the performance of iResNet is much better even though CRL method employs a more complex network structure.

Other methods try to integrate additional information to enhance the performance on these difficult regions. Xiao et al. [32] proposed a network composed of a backbone disparity network and an edge subnetwork. This model integrates edge cues by featuring embedding and edge-aware smoothness loss regularization and thus results in state-of-the-art performance on both KITTI stereo and scene flow benchmarks. Guorun et al. [25] proposed a model that integrates semantic features from segmentation and introduced the semantic softmax loss. The incorporation of the semantic cues greatly improved the prediction in disparity estimation and achieved state-of-the-art results on KITTI stereo benchmarks.

Unlike DispNet and its variants, several methods focus on designing a powerful regularization module based on 3D convolution [5, 28, 60, 61], as shown in Figure 4(b). Kendall et al. proposed the GC-Net [60] and were the first to use 3D convolution networks to aggregate context for cost volumes. Instead of collapsing the feature dimension when computing the cost volume, they formed a 4D cost volume with concatenated features from the image pairs along the disparity dimension followed by 3D convolution networks to give the disparity prediction. The usage of the disparity dimension greatly improved the performance and achieved state-of-the-art performance. Inspired by GC-Net, Chang and Chen [28] proposed the pyramid stereo matching network (PSMNet) to exploit the global context information. This network consists of spatial pyramid pooling and stacked 3D CNN modules. The spatial pyramid pooling extracts multiscale representations, and stacked 3D CNN regularizes the 4D cost volume to give the disparity prediction. This method ranked first in the KITTI 2012 and 2015 leaderboards before March 18, 2018.

Though these end-to-end deep learning networks recently demonstrated extremely good performance for stereo matching, they may suffer from the memory usage and low speed due to the 3D convolution process. Take GC-Net as an example: it takes about 10.4G GPU memory when processing a 1216 × 352 image pair [5]. To address this problem, Lu et al. proposed the sparse cost volume net (SCV-Net) [5] based on GC-Net. A stride was introduced when generating cost volume from features of image pair and the batch size and disparity dimensions were merged to make a 4D cost volume. This design greatly reduces the memory usage without compromising the performance. Tulyakov et al. [61] designed a practical deep stereo (PDS) network. The memory footprint was reduced by introducing a novel bottleneck matching module, which compresses left-right concatenated image descriptors into compact matching representations.

Besides these two popular structures, some works focus on designing specific functional modules. Lidong et al. [30] proposed a learning-based cost aggregation method for better generation and selection of cost aggregation proposals from cost volumes by a novel subarchitecture in the end-to-end trainable pipeline. This two-stream network offers global view guidance for the cost aggregation and reaches state-of-the-art performance on KITTI benchmarks. Jie et al. [33] proposed a novel left-right comparative recurrent (LRCR) model to perform left-right consistency checks jointly with an end-to-end disparity estimation network using stacked convolutional LSTM, upon which disparity maps are progressively improved. This approach achieves state-of-the-art result on KITTI benchmarks. However, the LSTM structure is much more time consuming compared to traditional CNN. As a result, this method is very time consuming as shown in Table 7. Poggi et al. [62] proposed a confidence measurement network to estimate the reliability of the predicted disparity. Slossberg et al. [63] introduced a densely connected conditional random field (CRF) which provides the a priori knowledge of interpixel interactions to regularize the cost volume. Kim et al. [64] present a deep architecture that estimates a stereo confidence.

End-to-end architectures achieve state-of-the-art results in disparity estimation, as listed in Tables 6 and 7. However, these methods still have difficulties finding correct correspondences in textureless regions, detailed structures, small objects, and near boundaries. Moreover, end-to-end stereo-matching-networks-based approaches generally require huge memory use, especially for the regularization modules composed of 3D convolutions. Even though several techniques have been employed, such as group-wise [31] and sparse technique [5], to address this problem, there is still a long way to go before a practical solution is developed. And of course, this kind of end-to-end stereo matching network needs corresponding ground truth depth data for training, which is a challenging problem.

## 4. Unsupervised Stereo Matching

Unsupervised stereo matching approaches rely on minimizing photometric warping error to drive the network in an unsupervised way. Over the past few years, based on spatial transformation and view synthesis, several unsupervised learning methods have been proposed.

Flynn et al. [34] introduced a novel image synthesis network called DeepStereo that generates new views by selecting pixels from nearby images. The Deep3D network by Xie et al. [37] also addressed the problem of novel view synthesis. In their method, the right view is generated from an input left image (i.e., the source image) in the context of binocular pairs by minimizing pixel-wise reconstruction loss. Again, using an image reconstruction loss, their method produces a distribution over all the possible disparities for each pixel. These view synthesis networks provide great support to unsupervised stereo matching. Based on Deep3D, Luo et al. reformulated the problem of monocular depth estimation into two subproblems, namely, a view synthesis procedure followed by a standard stereo matching. The main structure of the network is a combination of a Deep3D and a DispNet. The Deep3D provides the other view and DispNet predicts disparity from the initial image and the new view.

Garg et al. [35] proposed the first unsupervised network for single-view depth estimation using an image reconstruction loss. The network explicitly generates an inverse warp of the target image using the predicted depth to reconstruct the source image. Taylor expansion is employed to make the inverse warping differentiable and to make the training objective suboptimal. Even so, the network gives performance comparable to that of the state-of-the-art supervised methods for single-view depth estimation. However, due to the overall scale ambiguity from a single image, this monocular depth is not only inaccurate in an absolute sense, but also inaccurate in recovering details.

**Figure 5:**
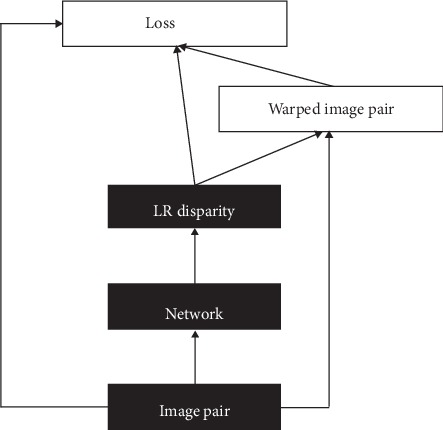
Standard pipeline of unsupervised stereo matching algorithms.

Ren et al. [65] adopted a bilinear sampling net to generate images, resulting in a fully differentiable training loss. Yu et al. [66] extended the image reconstruction loss together with a spatial smoothness loss for unsupervised optical flow learning. However, neither of them takes the advantage of geometric consistency among predictions until Godard et al. [36]. Godard et al. demonstrated that the solvation of image reconstruction alone results in poor quality depth images. To address this problem, they proposed a network architecture with a novel training loss that enforces left-right depth consistency inside the unsupervised end-to-end network. The consistency constraint greatly improves the performance, even outperforming supervised methods that have been trained with ground truth depth. This work marked the maturity of the unsupervised stereo matching approaches which rely on minimizing photometric warping error. Several other approaches have been proposed based on this structure [36]. The standard pipeline of the unsupervised stereo matching is shown in Figure 5: (1) given an image pair, the network module outputs the left and right disparity maps; (2) warped image pair was generated based on the disparity maps and the origin image pair; (3) image reconstruction loss and LR-consistency loss are generated by the disparity maps, origin image pair, and warped image pair, making an end-to-end training framework. After training, the disparity prediction procedure is conducted as the black block pipeline shown in Figure 5. Based on this standard pipeline, Zhong et al. [39] proposed an unsupervised self-adaptive stereo matching network by combining two GC-Nets together, each of which produces a disparity estimation. The training loss in this network is similar to [36]. Smolyanskiy et al. [40] slightly changed the architecture and proposed a semisupervised approach where ground truth depths and unsupervised binocular alignment losses are both used to train the monocular depth estimation network 

There are other methods focusing on the optical flow estimation by incorporating the pose information. Zhou et al. [41] presented an unsupervised learning framework for the task of monocular depth and camera motion estimation. By using an end-to-end learning approach with view synthesis as the supervisory signal, the approach predicts the monocular depth and ego-motion in a coupled way. Similarly, Vijayanarasimhan et al. [67] proposed a geometry-aware neural network for motion estimation in videos that could learn depth, segmentation, camera, and rigid object motions together. Yin et al. [68] also proposed a jointly unsupervised learning framework for monocular depth, optical flow and ego-motion estimation. However, extending these monocular methods to stereo matching is nontrivial. When feeding the networks with stereo pairs, their performances are not even comparable to traditional stereo matching methods.

## 5. Conclusion

This article provides a comprehensive coverage of the remarkable stereo matching approaches based on deep learning. For convenience, we grouped these approaches into three categories: (1) non-end-to-end learning algorithms, (2) end-to-end learning algorithms, and (3) unsupervised learning algorithms.

The non-end-to-end framework has been thoroughly studied by previous researchers. Several works focus on calculating the similarity between two image patches to form cost volumes, while others try to substitute other components in the legacy stereo pipeline. Both of these fields achieved great success but still suffer from high computational burden, limited receptive field, and lack of context information, and they still use postprocessing functions more or less.

End-to-end approaches could achieve the state-of-the-art results due to their powerful representation ability. Moreover, end-to-end approaches provide a very convenient way to calculate disparity. Some works focus on designing new architecture and try their best to incorporate more context information to improve the quality of the disparity, especially in the textureless and occlusion region. A small group of researchers start to care about the speed and memory usage problem. And this problem will attract more and more researchers as the problem is ubiquitously existent in end-to-end usage methods, and it severely prevents these algorithms from practical usage in embedded devices.

The unsupervised methods aim to solve the label burden and achieved great progress. However, the existing methods still suffer from low quality of the results. This is mainly because the image reconstruction error could not provide a very powerful strength to let the network converge to the ground truth disparity. And the left-right consistency error intrinsically damaged the correctness around the occlusion area. Therefore, it is attracting more and more researchers, making it a hot topic in the stereo matching field.

## Figures and Tables

**Table 1:** Comparison of the three frameworks.

Framework	Inst.	Advantage	Disadvantage
Non-end-to-end	MC-CNN, content-CNN, SGM-Net	(1) Simple; (2) better performance compared to traditional methods	(1) High computational burden; (2) limited receptive field and the lack of context information; (3) still using postprocessing
End-to-end	PSMNet, GC-Net	(1) Disparity image quality; (2) easy to design	(1) Huge cost burden and large memory footprint; (2) long time cost; (3) needing ground truth data
Unsupervised	LR-consistency-check [36]	(1) Not needing ground truth data	(1) Poor performance

**Table 2:** Comparison of unsupervised stereo matching methods on the KITTI stereo 2015 benchmark.

Method	Abs rel	Sq rel	RMSE	RMSE log	*δ* < 1.25 (%)	*δ* < 1.25^2^ (%)	*δ* < 1.25^3^ (%)	Runtime (s)	Environment
Luo et.al [42]	0.094	**0.626**	**4.252**	0.177	0.891	0.965	0.984	—	—
Garg et al. [35]	0.169	1.080	5.104	0.273	0.740	0.904	0.962	—	—
Godard et al. [36]	**0.068**	0.835	4.392	**0.146**	**0.942**	**0.978**	**0.989**	0.035	Nvidia Titan X
Zhou et al. [41]	0.208	1.768	6.856	0.283	0.678	0.885	0.957	—	—
Yin and Shi [38]	0.155	1.296	5.857	0.233	0.793	0.931	0.973	**0.015**	Nvidia Titan X

**Table 3:** Comparison of stereo matching methods using CNN for cost calculation on the KITTI stereo 2015 benchmark.

Methods	>2 pixels		>3 pixels		>4 pixels		>5 pixels		Mean error		Runtime (s)	Environment
	Non-occ	All	Non-occ	All	Non-occ	All	Non-occ	All	Non-occ	All		
Deep Embed [19]	5.05	6.47	3.10	4.24	2.32	3.25	1.92	2.68	0.9 px	1.1 px	3	Nvidia GTX Titan (CUDA, Caffe)
MC-CNN-acrt [16]	**3.90**	**5.45**	**2.43**	**3.63**	**1.90**	**2.85**	**1.64**	**2.39**	**0.7 px**	0.9 px	67	Nvidia GTX Titan (CUDA, Lua/Torch7)
Content-CNN [17]	4.98	6.51	3.07	4.29	2.39	3.36	2.03	2.82	0.8 px	1.0 px	**0.7**	Nvidia Titan X (CUDA)
OCV-SGBM	9.47	10.86	—	—	—	—	—	—	—	—	1.1s at 2.5 GHz CPU	2.5 GHz CPU (C++)

**Table 4:** Comparison of non-end-to-end stereo matching methods using CNN for cost aggregation and postprocessing on the KITTI stereo 2012 benchmark.

Methods	>2 pixels (%)		>3 pixels (%)		>4 pixels (%)		>5 pixels (%)		EPE NOC (px)	Runtime (s)	Environment
	Non-occ	All	Non-occ	All	Non-occ	All	Non-occ	All			
SGM-NET [20]	**3.60**	5.15	**2.29**	3.50	**1.83**	2.80	**1.60**	2.36	0.7 px	**67**	Nvidia (R) Titan X (Torch7)
Displets [52]	3.90	**4.92**	2.37	**3.09**	1.97	**2.52**	1.72	**2.17**	0.7 px	265	8+ cores at 3.0 GHz (Matlab + C/C++)

**Table 5:** Comparison of non-end-to-end stereo matching methods using CNN for cost aggregation and postprocessing on the KITTI stereo 2012 benchmark.

Methods	All pixels			Nonoccluded pixels			Runtime (s)	Environment
	D1-bg (%)	D1-fg (%)	D1-all (%)	D1-bg (%)	D1-fg (%)	D1-all (%)		
Displets [52]	3.00	**5.56**	3.43	2.73	4.95	3.09	265	8+ cores @ 3.0 GHz (Matlab + C/C++)
SGM-Net [20]	2.66	8.64	3.66	**2.23**	7.44	3.09	67	Nvidia (R) Titan X (Torch7)
DRR [22]	**2.58**	6.04	**3.16**	2.34	**4.87**	**2.76**	**0.4**	Nvidia (R) Titan X (--)
CNN + CRF [21]	—	—	5.50	—	—	4.84	1.3 s	C++/CUDA

**Table 6:** Comparison of end-to-end stereo matching methods on the KITTI stereo 2012 benchmark.

Methods	>2 pixels (%)		>3 pixels (%)		>4 pixels (%)		>5 pixels (%)		EPE NOC	Runtime (s)	Environment
	Non-occ	All	Non-occ	All	Non-occ	All	Non-occ	All			
PSMNet [28]	2.44	3.01	1.49	1.89	1.12	1.42	0.90	1.15	0.5 px	0.41	Nvidia Titan Xp (CUDA)
SegStereo [25]	2.66	3.19	1.68	2.03	1.25	1.52	1.00	1.21	0.5 px	0.6	Caffe
iResNet [23]	2.69	3.34	1.71	2.16	1.30	1.63	1.06	1.32	0.5 px	0.12	Nvidia Titan
X (Caffe)											
GC-Net [60]	2.71	3.46	1.77	2.30	1.36	1.77	1.12	1.46	0.6 px	0.9	Nvidia Titan
X (--)											
PDSNet [61]	3.82	4.64	1.92	2.53	1.38	1.85	1.12	1.51	0.9 px	0.5	Nvidia Titan X
L-ResMatch [18]	3.64	5.06	2.27	3.40	1.76	2.67	1.50	2.26	0.7 px	48	Nvidia Titan X
DispNet [24]	7.38	8.11	4.11	4.65	2.77	3.20	2.05	2.39	0.9 px	**0.06**	Nvidia Titan X
EdgeStereo [32]	2.32	2.88	1.46	1.83	**1.07**	**1.34**	0.83	1.04	**0.4 px**	0.32	Nvidia GTX 1080Ti (Caffe)
GwcNet-gc [31]	**2.16**	**2.71**	**1.32**	**1.70**	—	—	**0.80**	**1.03**	0.5 px	0.32	Nvidia Titan Xp (-)

**Table 7:** Comparison of end-to-end stereo matching methods on the KITTI stereo 2015 benchmark.

Methods	All pixels			Non-occluded pixels			Runtime (s)	Environment
	D1-bg (%)	D1-fg (%)	D1-all (%)	D1-bg (%)	D1-fg (%)	D1-all (%)		
PSMNet [28]	1.86	4.62	2.32	1.71	4.31	2.14	0.41	Nvidia Titan Xp (CUDA)
SegStereo [25]	1.88	4.07	2.25	1.76	3.70	2.08	0.6	Caffe
iResNet [23]	2.25	3.40	2.44	2.07	**2.76**	2.19	0.12	Nvidia Titan X (Caffe)
GC-Net [29]	2.21	6.16	2.87	2.02	5.58	2.61	0.9	Nvidia Titan X (--)
PDSNet [61]	2.29	4.05	2.58	2.09	3.68	2.36	0.5	Nvidia Titan X
L-ResMatch [18]	2.72	6.95	3.42	2.35	5.74	2.91	48	Nvidia Titan X
EdgeStereo [32]	1.84	**3.30**	**2.08**	1.69	2.94	**1.89**	0.32	Nvidia GTX 1080Ti (Caffe)
CRL [26]	2.48	3.59	2.67	2.32	3.12	2.45	0.47	Nvidia GTX 1080
LRCR [33]	2.55	5.42	3.03	2.23	4.19	2.55	49.2	--
DispNet [24]	4.32	4.41	4.34	4.11	3.72	4.05	**0.06**	Nvidia Titan X
GwcNet-gc [31]	**1.74**	3.93	2.11	**1.61**	3.49	1.92	0.32	Nvidia Titan Xp (--)
SCV-Net [5]	2.22	4.53	2.61	2.04	4.28	2.41	0.36	Nvidia GTX 1080Ti
